# The Relationship between Health Literacy and Health Behaviour in People with Diabetes: A Danish Population-Based Study

**DOI:** 10.1155/2016/7823130

**Published:** 2016-09-28

**Authors:** Karina Friis, Benedicte Donslund Vind, Rebecca K. Simmons, Helle Terkildsen Maindal

**Affiliations:** ^1^DEFACTUM, Public Health & Health Services Research, Central Denmark Region, Olof Palmes Allé 15, 8200 Aarhus N, Denmark; ^2^Department of Public Health, Section of General Practice, Aarhus University, Aarhus, Denmark; ^3^Danish Diabetes Academy, Odense, Denmark; ^4^Medical Research Council Epidemiology Unit, University of Cambridge School of Clinical Medicine, Cambridge, UK; ^5^Steno Diabetes Center, SCHP, Niels Steensens Vej 6, Gentofte, Denmark; ^6^Department of Public Health, Aarhus University, Bartholins Allé 2, 8000 Aarhus C, Denmark

## Abstract

*Background*. People with diabetes who have poor health behaviours are at greater risk for a range of adverse health outcomes. We aimed to investigate the relationship between health literacy and health behaviour (smoking, alcohol, physical activity, and diet) in people with diabetes.* Methods*. The study was based on respondents aged 25 years or older from a population-based survey in 2013 who reported having diabetes (*n* = 1685). Two dimensions from the Health Literacy Questionnaire were used: “understand health information” and “actively engage with healthcare providers.” We used logistic regression to examine the association between health literacy and health behaviour.* Results*. After adjustment for sociodemographic factors, individuals with diabetes who found it difficult to understand information about health had higher odds of being physically inactive (OR: 3.43, 95% CI: 2.14–5.51) and having unhealthy dietary habits (OR: 3.01, 95% CI: 1.63–5.58). Similar results were observed for individuals who found it difficult to actively engage with healthcare providers. No associations were found between the two dimensions of health literacy and smoking and alcohol consumption.* Conclusion*. When developing health services and interventions to improve health behaviour among people with diabetes, our results suggest that they may benefit by including focus on health literacy.

## 1. Background

People with diabetes are at risk for a range of adverse health outcomes, including heart attacks, strokes, amputations, blindness, and end-stage renal disease [1]. Many of these adverse health outcomes can be prevented or delayed if people with diabetes maintain a healthy lifestyle in relation to diet, physical activity, alcohol, and smoking [2, 3]. Thus, it is important for health services and healthcare providers to develop strategies and interventions to help people with diabetes to improve and maintain their health behaviour.

Health literacy is defined as the cognitive and social skills that determine a person's motivation and ability to gain access to, understand, and use information in ways that promote and maintain good health [4]. Health literacy brings together many concepts that relate to what people need in order to make effective decisions about health for themselves and their families. A recent Danish population-based study has shown that, even after adjusting for sociodemographic factors and comorbidities, people with diabetes find it more difficult to understand health information than the general population [5].

Diabetes is a chronic disease characterized by a high level of complexity that requires extensive self-care management including translation of guidelines into everyday life [6]. The demands for people with diabetes are complicated because self-care of a chronic disease often relies on information in printed educational materials, verbal instructions, and patient education courses [7]. People with low levels of health literacy may struggle to find and follow these instructions, when they are to be integrated in everyday life. Furthermore, low levels of health literacy are associated with poor glycaemic control in diabetes patients [8], as well as with a number of diabetic complications [9]. Therefore, having adequate health literacy is critical for diabetes patients for managing their condition and navigating the healthcare environment.

Some studies have shown that inadequate health literacy is associated with unhealthy behaviours such as smoking, physical inactivity, and poor diet in the general population [10–14], while one study [15] shows that health literary is not independently associated with some health behaviours. Few studies have investigated the relationship between health literacy and health behaviour such as smoking, alcohol consumption, physical activity, and diet in people with diabetes [16–20]. These studies showed no association between health literacy and these health behaviours. However, these studies were all conducted in small clinical settings and only measured health literacy in terms of cognitive and functional skills such as reading ability. To the best of our knowledge, no studies have investigated the association between health literacy and health behaviour in people with diabetes using a more comprehensive measure of health literacy including social and communication skills.

Using data from a large population-based survey, we aimed to investigate the association between health literacy and health behaviour (smoking, alcohol consumption, physical activity, and diet) in people with diabetes.

## 2. Methods

### 2.1. Study Design and Data Collection

The study is based on data from respondents aged 25 years or older from the 2013 Danish health and morbidity survey called “How Are You?” Geographically, Denmark is divided into five administrative regions and this study comprises data from one of these regions, the Central Denmark Region, where approximately 22% of the Danish population lives. Regarding sociodemographic and health related factors, the population of the Central Denmark Region is similar to the whole Danish population [21].

The survey consisted of a random sample of 46,354 people who were drawn from the Danish Civil Registration System. People were invited to complete a postal or a web-based questionnaire. Three reminders were issued. Data were collected by the Central Denmark Region between February and April 2013. In total, 29,473 people (63.6%) completed and returned the questionnaire. The questionnaire included an item on diabetes status; 1,685 individuals (5.7%) reported having diabetes.

### 2.2. Measures

#### 2.2.1. Health Literacy

The Health Literacy Questionnaire (HLQ) is a widely used measure of health literacy that has been translated into many European and Asian languages [22]. The HLQ consists of nine dimensions and was developed using a validity-driven approach including in-depth grounded consultations, psychometric analyses, and cognitive interviews [22]. The translation and cultural adaption of the questions from English into Danish followed a rigorous forward-backward translation procedure and cognitive testing to ensure cross-cultural validity [23].

In the health and morbidity survey, two of the nine HLQ dimensions were included: “understand health information well enough to know what to do” to measure the functional dimension and “actively engage with healthcare providers” to measure the communicative dimension. Given that population surveys have limited space for survey questions, only these two scales were selected from the HLQ. The two scales cover two distinct elements of health literacy which we hypothesized would provide valuable insight into the health literacy challenges of individuals with chronic diseases. Each scale comprised five items where participants indicated their response on a four-point scale: 1 = very difficult, 2 = difficult, 3 = easy, and 4 = very easy. Scale scores were calculated as the mean of the five-item scores and then standardized to range between 1 (lowest ability) and 4 (highest ability) to ensure consistency with the response options. If responses to more than two items on a scale were missing for an individual, the scale score for that individual was regarded as missing. As a result of this, 137 observations (7.5%) were excluded from the “understand health information” scale and 131 observations (7.2%) from the “actively engage with healthcare providers” scale. Cronbach's alpha coefficients indicated that the internal consistency of both scales was high: “understand health information” *α* = 0.86 and “actively engage with healthcare providers” *α* = 0.90. The scales correlated positively with one another (Pearson's coefficient = 0.78). We dichotomised the scale to identify individuals who found it very difficult or difficult (score ≤ 2) to understand health information or to actively engage with healthcare providers.

#### 2.2.2. Health Behaviour

Four measures of health behaviour (smoking, alcohol consumption, physical inactivity, and unhealthy diet) were used. Respondents who indicated that they smoked on a daily basis were classified as smokers. Respondents were asked how many alcoholic drinks per week they normally drink. High-risk alcohol consumption was categorized in accordance with the Danish Health Authority's recommendations, that is, more than 21 drinks weekly for men and 14 drinks for women. Respondents were classified as physically inactive if, during a typical week, they were not physically active at least one day for a minimum of 30 minutes. Dietary habits were assessed using the validated Diet Quality Score [24], which classifies diet quality in relation to cardiovascular risk. The scale consists of 25 items including questions about type of bread spread, fats used for cooking, and how often the participants consumed selected food items (including fish, meat, fruits, and vegetables). The diet score was calculated and categorized into two groups: unhealthy diet and very healthy/reasonably healthy diet. Unhealthy diet was defined as having low intake of fruit, vegetables, and fish and a high amount of saturated fat [24].

#### 2.2.3. Demographic and Socioeconomic Factors

Data on age, gender, ethnic background, and marital status were collected from national registers to achieve complete data. Respondents were defined as Danish if they had Danish citizenship or if at least one of their parents was a Danish citizen. Marital status refers to whether an individual is married or not. Information about educational attainment was derived from survey data. The participants were asked about their highest level of completed school education and any further higher-level education. We categorized educational attainment as low (1–10 years), medium (11–14 years), and high (≥15 years).

### 2.3. Ethics

The study was approved by the Danish Data Protection Agency and was conducted in accordance with the Helsinki Declaration. Information about the survey was provided to potential participants in writing and via the web. The participants' voluntary completion and return of the survey questionnaires constituted implied consent.

### 2.4. Statistical Analysis

The unique personal identification number given to all Danish citizens was used to link both respondents and nonrespondents to Danish national registers. A weight was constructed using a model-based calibration approach based on register information from Statistics Denmark. The weight accounted for differences in selection probabilities and response rates between subgroups. Data was weighted to represent the population of the Central Denmark Region and was used in all the data analyses.

To examine the association between health literacy and health behaviour in people with diabetes, eight logistic regression models were conducted, one for each health literacy dimension with the four different health behaviour measures (daily smoking, high-risk alcohol consumption, physical inactivity, and unhealthy diet) as dependent variables. In each logistic regression model, the odds ratios were further adjusted for gender, age, ethnic background, educational attainment, and marital status.

Significance was set at *p* < 0.05. Statistical analyses were performed using STATA 13.

## 3. Results

Table 1 describes participant characteristics in relation to sociodemographic factors, the two health literacy dimensions, and health behaviour. Of the 1,685 individuals with diabetes, 34.1% had a low level of education. The majority of the participants were of Danish origin. In total, 9.3% of the participants found it difficult or very difficult to understand health information, and 9.3% found it difficult or very difficult to actively engage with healthcare providers. 11.8% of our sample had difficulties on at least one of the two scales (data not shown). The health behaviour characteristics of the participants show that 21.1% were daily smokers, 6.5% had high-risk alcohol consumption, 30.7% were physically inactive, and 12.3% had unhealthy dietary habits.

Nonresponse in the ten health literacy items was low and evenly distributed (between 5.3% and 8.2%) (Table 2), suggesting that the items were understood and had acceptable content. For all items, all response options were endorsed by some respondents although there were fewer in the extreme “very difficult” category and many in the “easy” category (Table 2).

Table 3 describes the association between health literacy and health behaviour in people with diabetes. After adjusting for gender, age, ethnic background, educational affiliation, and cohabitation status, people who found it difficult to understand information about health had higher odds of being physically inactive (OR: 3.43, 95% CI: 2.14–5.51) and having unhealthy dietary habits (OR: 3.01, 95% CI: 1.63–5.58). Similarly, people who found it difficult to actively engage with healthcare providers had higher odds of being physically inactive (OR: 2.72, 95% CI: 1.76–4.20) and having unhealthy dietary habits (OR: 2.73, 95% CI: 1.51–4.94). No significant results were found for the association between the two dimensions of health literacy and cigarette smoking and alcohol consumption.

## 4. Discussion

Results from this large population-based survey suggest that 9% of the participants found it difficult or very difficult to understand health information, and 9% found it difficult or very difficult to actively engage with healthcare providers. Respondents who found it difficult to understand information about health had higher odds of being physically inactive and having unhealthy dietary habits. Similar results were seen for people who found it difficult to actively engage with healthcare providers.

For diabetes patients with low health literacy levels, it may be difficult to navigate the large number of recommendations on diet and physical activity behaviour. These are complex behaviours that everyone uses on a daily basis and are subject to a number of individual and societal pressures that may be difficult to change. On the contrary, recommendations about smoking and alcohol consumption are generally more straightforward and have been promoted for several decades now. For example, the Danish Health Authority has run several antismoking campaigns and Denmark has continually undergone legislative changes with regard to smoking during the last decade, for example, tax on cigarettes and smoking bans at restaurants and public areas [25]. This attention to smoking may have led to high awareness about the risk of smoking in the Danish population and also among people with diabetes, and therefore information on smoking risk might be easier to understand, regardless of health literacy level compared with other health behaviours.

In contrast with our results, most research in individuals with diabetes does not support an association between health literacy and health behaviour such as physical activity and dietary habits [16–20]. However, research on health literacy in people with diabetes has focused on a one-dimensional concept of health literacy, that is, verbal ability. Furthermore, research has been conducted in clinical settings making it difficult directly to compare our results with other studies. For example, Bains and Egede showed no association between health literacy and physical activity and diet [16]. However, their study only included 125 adults recruited from a primary care clinic in the United States. Additionally, they assessed health literacy by asking patients to pronounce medical words, thus having a more narrow measure of health literacy than in our study. Kim et al. also found no association between health literacy and health behaviour, but they too had a small clinical sample consisting of 92 patients and the researchers only measured reading abilities [17]. In another study, smoking, physical activity, and diet were not significantly associated with health literacy [18]. These results on smoking are similar to our study. However, only 50 African Americans participated in the study, and health literacy was measured in terms of pronunciation and reading ability.

### 4.1. Implications

People with diabetes often have an ongoing interaction with the healthcare system and meet many healthcare practitioners throughout the life course. The challenges of adhering to public health recommendations concerning diet and physical activity are well known, particularly among patients with long-term conditions such as diabetes. Patients with long-term conditions such as diabetes need support to develop and maintain their health literacy skills. Our study suggests that it is difficult for patients with diabetes and low health literacy levels to adhere to recommended treatment guidelines. Adequate health literacy is crucial for patients to make optimal choices for their health. Healthcare providers therefore need to be aware of health literacy oriented strategies to support patients in making such choices. One strategy is to educate healthcare providers to communicate health information so that it is tailored to develop patients' understanding of their health condition and how to manage it. Exploring health literacy levels in more detail among individuals with diabetes with newly developed and validated tools is also a promising avenue of research [26].

### 4.2. Strengths and Limitations

We used data from a large population-based survey with a high response rate. As the survey was not focussed on individuals with diabetes, this may have lowered the risk of social desirability bias when responding to questions on health behaviour and health literacy level. An advantage of using a population-based sample for this study was that we included diabetes patients in the long maintenance phase of living with the disease. Many clinical studies only include individuals at the time of diagnosis or when adverse health outcomes cause them to use the healthcare services. The self-reported prevalence of diabetes was 5.7% in our study sample. This agrees well with data from the Danish National Diabetes Register [27], which shows that 6% of the Danish population above the age of 16 has diabetes. Another strength of this study was that we had the opportunity to control for a wide range of sociodemographic factors. Furthermore, to date, most health literacy research has focused on reading ability and numeracy based on data collected through direct testing procedures [28–30]. We used two different self-reported dimensions of health literacy that capture a dynamic state depending on how the individual person perceives his or her current situation.

Our findings are based on cross-sectional data, and therefore no conclusions about the temporality or causation can be made. Also, we were unable to differentiate between individuals with type 1 and type 2 diabetes. Health literacy and health behaviours might be different in these two groups. Also, it should be noted that there may be some imprecision and bias associated with using self-report measures of behaviour. Furthermore, the ability and motivation to fill out a health survey may be viewed as a health literacy competency in itself; thus, the most vulnerable groups may have been excluded from our study. As the questionnaire was not translated into other languages, people who had limited Danish language skills may not have participated in the survey. The study is also limited by including only two of the nine defined dimensions of the HLQ. Thus, it suffers from construct underrepresentation [31]. We can therefore draw conclusions only about the two dimensions we measured and not about health literacy overall. Application of the complete tool was not possible for practical reasons in this large population survey. We dichotomised the health literacy dimensions to be able to differentiate between respondents who found it “difficult” and “easy” to understand health information. This may have reduced the power to explore potential associations. However, using the exposure variable as a continuous measure did not change the overall results.

## 5. Conclusion

Even after adjusting for sociodemographic factors, people with diabetes who find dimensions of health literacy difficult have higher odds of being physically inactive and having unhealthy dietary habits compared to people who do not have these difficulties. Strategies for improving physical activity and diet among people with diabetes may benefit by having focus on health literacy within prevention, patient education, and other public health interventions.

## Figures and Tables

**Table 1:** Characteristics of participants with diabetes from the “How Are You?” survey, Central Denmark Region (2013) (*N* = 1,685).

	*N*	%
*Sociodemographic factors*		
Gender		
Male	954	54.9
Female	731	45.1
Age (years)		
25–44	115	9.5
45–64	652	39.5
65–84	866	47.1
85+	52	4.0
Educational attainment		
Low	529	34.1
Medium	790	48.9
High	282	17.1
Ethnic background		
Danish	1633	95.0
Non-Danish	52	5.0
Marital status		
Living alone	479	38.4
Married/cohabiting	1170	61.6
*Health literacy dimensions*		
Understand health information		
Difficult/very difficult	121	9.3
Easy/very easy	1446	90.7
Mean scale score (2.92, SD 0.61)		
Actively engage with healthcare providers		
Difficult/very difficult	133	9.3
Easy/very easy	1438	90.7
Mean scale score (3.00, SD 0.62)		
*Health behaviours*		
Daily smoker		
No	1319	78.9
Yes	330	21.1
High-risk alcohol consumption^1^		
No	1427	93.5
Yes	102	6.5
Physically inactive^2^		
No	1172	69.3
Yes	457	30.7
Unhealthy dietary habits^3^		
No	1390	87.7
Yes	168	12.3

^1^≥21 drinks/week for men and ≥14 drinks/week for women.

^2^Max. 30 minutes of physical activity one day during a typical week.

^3^Low intake of fruit, vegetables, and fish, and a high amount of saturated fat.

**Table 2:** Response distribution and missing values for each item of the two health literacy scales.

	Very easy (%)	Easy (%)	Difficult (%)	Very difficult (%)	Item missing (%)
*Understanding health information well enough to know what to do*					
Confidently fill in medical forms in the correct way	19.9	50.6	16.3	5.8	7.4
Accurately follow the instructions from healthcare providers	17.0	51.8	20.2	2.9	8.2
Read and understand written health information	19.1	53.6	15.6	4.7	6.8
Read and understand all the information on medication labels	16.9	49.7	20.2	6.9	6.3
Understand what healthcare providers are asking you to do	19.8	59.8	11.0	2.5	7.0
*Ability to actively engage with healthcare providers*					
Make sure that healthcare providers understand your problems properly	20.2	49.1	19.5	3.4	7.9
Feel able to discuss your health concerns with a healthcare provider	23.3	53.8	14.6	2.0	6.4
Have good discussions about your health with doctors	25.0	53.6	13.5	2.5	5.3
Discuss things with healthcare providers until you understand all you need to	19.6	51.8	17.1	3.7	7.8
Ask healthcare providers questions to get the health information you need	20.5	54.0	14.9	3.4	7.6

**Table 3:** Association between health literacy dimensions and health behaviour among individuals with diabetes from the “How Are You?” survey, Central Denmark Region (2013) (*N* = 1,685).

	Daily smoking		High-risk alcohol consumption		Physically inactive		Unhealthy dietary habits	
	Unadjusted OR (95% CI)	Adjusted OR^a^ (95% CI)	Unadjusted OR (95% CI)	Adjusted OR^a^ (95% CI)	Unadjusted OR (95% CI)	Adjusted OR^a^ (95% CI)	Unadjusted OR (95% CI)	Adjusted OR^a^ (95% CI)
*Understand health information*								
Difficult/very difficult	0.94 (0.55–1.60)	0.87 (0.47–1.59)	1.56 (0.76–3.19)	2.09 (0.90–4.81)	4.50^*∗*^ (2.85–7.11)	3.43^*∗*^ (2.14–5.51)	3.11^*∗*^ (1.79–5.40)	3.01^*∗*^ (1.63–5.58)
Easy/very easy	1	1	1	1	1	1	1	1
*Actively engage with healthcare providers*								
Difficult/very difficult	1.07 (0.64–1.80)	1.03 (0.59–1.79)	1.18 (0.54–2.58)	1.45 (0.63–3.34)	2.83^*∗*^ (1.85–4.34)	2.72^*∗*^ (1.76–4.20)	2.59^*∗*^ (1.48–4.52)	2.73^*∗*^ (1.51–4.94)
Easy/very easy	1	1	1	1	1	1	1	1

^a^OR adjusted for gender, age, ethnic background, educational attainment, and marital status.

^*∗*^ *p* < 0.05.

CI: confidence interval.
